# Immunomodulation Stimulates the Innervation of Engineered Tooth Organ

**DOI:** 10.1371/journal.pone.0086011

**Published:** 2014-01-22

**Authors:** Tunay Kökten, Thibault Bécavin, Laetitia Keller, Jean-Luc Weickert, Sabine Kuchler-Bopp, Hervé Lesot

**Affiliations:** 1 Institut National de la Santé Et de la Recherche Médicale (INSERM) Unité Mixte de Recherche (UMR)1109, team “Osteoarticular and Dental Regenerative NanoMedicine”, Faculté de Médecine, Université de Strasbourg, Strasbourg, France; 2 Faculté de Chirurgie Dentaire, Université de Strasbourg, Strasbourg, France; 3 Service de Microscopie Electronique, Institut de Génétique et de Biologie Moléculaire et Cellulaire (IGBMC), INSERM Unité (U)964, Centre National de la Recherche Scientifique (CNRS) UMR1704, Université de Strasbourg, Illkirch, France; Université de Technologie de Compiègne, France

## Abstract

The sensory innervation of the dental mesenchyme is essential for tooth function and protection. Sensory innervation of the dental pulp is mediated by axons originating from the trigeminal ganglia and is strictly regulated in time. Teeth can develop from cultured re-associations between dissociated dental epithelial and mesenchymal cells from Embryonic Day 14 mouse molars, after implantation under the skin of adult ICR mice. In these conditions however, the innervation of the dental mesenchyme did not occur spontaneously. In order to go further with this question, complementary experimental approaches were designed. Cultured cell re-associations were implanted together with trigeminal ganglia for one or two weeks. Although axonal growth was regularly observed extending from the trigeminal ganglia to all around the forming teeth, the presence of axons in the dental mesenchyme was detected in less than 2.5% of samples after two weeks, demonstrating a specific impairment of their entering the dental mesenchyme. In clinical context, immunosuppressive therapy using cyclosporin A was found to accelerate the innervation of transplanted tissues. Indeed, when cultured cell re-associations and trigeminal ganglia were co-implanted in cyclosporin A-treated ICR mice, nerve fibers were detected in the dental pulp, even reaching odontoblasts after one week. However, cyclosporin A shows multiple effects, including direct ones on nerve growth. To test whether there may be a direct functional relationship between immunomodulation and innervation, cell re-associations and trigeminal ganglia were co-implanted in immunocompromised Nude mice. In these conditions as well, the innervation of the dental mesenchyme was observed already after one week of implantation, but axons reached the odontoblast layer after two weeks only. This study demonstrated that immunodepression *per se* does stimulate the innervation of the dental mesenchyme.

## Introduction

Teeth can develop from cultured and implanted re-associations between dissociated dental epithelial and mesenchymal cells from Embryonic Day (ED) 14 mouse molars. In these experimental conditions, it is possible to reproduce the epithelial-mesenchymal interactions, which control odontogenesis during embryonic development. This approach allows the progressive steps involved in odontogenesis to proceed: crown morphogenesis, epithelial histogenesis, the initiation of root formation and the functional differentiation of odontoblasts, ameloblasts and cementoblasts [for review see [1]]. These steps were completed already after two weeks of implantation under the skin of adult ICR mice [2]. Other non-dental cell types have also been successfully used to replace either the mesenchymal [3] or epithelial dental embryonic cells [4]–[7]. In these conditions however, the other interacting tissue, the inductor, still needs to have a dental origin.

Still, very little is known about the innervation of engineered teeth. The sensory innervation of the dental mesenchyme is essential for tooth protection. Together with the innervation of the periodontium, it is also a key parameter for tooth function, by the perception of mechanical stress. Since odontoblasts are mechanosensory cells, the presence of nerve fibers in this cell layer is particularly important. Nevertheless, the way odontoblasts interact with and signal to axons is still unclear [8]. The presence of different types of glial cells next to the odontoblast layer and their relationship with microvascularization have been investigated in detail recently and illustrate the complexity of the cellular network involved in the process [9]. Sensory innervation of the dental pulp is mediated by axons originating from the trigeminal ganglion and is strictly regulated in time [10], [11]. The late innervation of the dental pulp during development, despite the presence of neurotrophic factors in it, suggested the involvement of inhibitory molecules at early stages [12], [13].

The innervation of the dental mesenchyme did not occur spontaneously when implanting cultured dental cell re-associations under the skin of adult ICR mice [1]. A specific study was thus developed, aiming to go further in detail with this question. For that purpose, complementary experimental approaches have been designed, using mouse dental embryonic cells [2]. Cultured dental cell re-associations were implanted under the skin of adult ICR mice for one or two weeks, either alone or together with trigeminal ganglia and used as control experiments. In the clinical context of face transplantation, it had been shown that immunosuppressive therapy accelerated the innervation of transplanted tissues [14]. cyclosporin A (CsA), which reversibly inhibits T-lymphocyte function, has been widely used in organ transplantation [15]. Co-implantations of cultured dental cells with trigeminal ganglia were thus performed in CsA-treated ICR mice. However, CsA also has direct effect on nerve growth [16]. To check whether immunosuppresion itself could interfere with the innervation of dental tissues, co-implantations were performed in Nude mice. The results demonstrated that immunodepression *per se* stimulates the innervation of the dental mesenchyme.

## Materials and Methods

### Animals and tissues

All procedures were designed in compliance with the recommendations of the European Union (2010/63/EU) for the care and use of laboratory animals. ICR mice (Charles River Laboratories, l′Arbresle, France) were mated overnight and the detection of the vaginal plug was determined as Embryonic Day (ED) 0. First lower molars were dissected from embryos at ED14 and trigeminal ganglia were dissected under a stereomicroscope (Leica MZ95, Nanterre, France) from newborn ICR mice PostNatal day (PN) 0 to 3. For immunosuppression, CsA (Néoral® Novartis, Rueil-Malmaison, France) was added to drinking water (1.2 ml Néoral®/L), one week before and all along the implantation period. The water containing CsA was changed every two days.

### Ethics statement

Experiments followed current European Union regulations (Directive 2010/63/EU), and were performed according to authorized investigator Dr. N. Jessel (Director of the «Osteoarticular and Dental Regenerative Nanomedicine» Team), holder of a personal license from «Préfecture du Bas-Rhin» (No. 67-315), who oversaw experiments done on mice.

All experiments were realized in the “Animalerie Centrale de la Faculté de Médecine de Strasbourg” with the approval number: A 67-482-35 from the Veterinary Public Health Service of the “Préfecture du Bas-Rhin”, representing the French Ministry of Agriculture, Department of Veterinary Science.

ICR mice (Charles River Laboratories, l′Arbresle, France) were mated overnight and the detection of the vaginal plug was determined as Embryonic Day (ED) 0. First lower molars for dental cell re-associations were all dissected from embryos at embryonic day 14. For this, pregnant mice were injected with a sub-lethal dose of pentobarbital (Centravet, Nancy, France) and embryos were delivered by caesarean section and decapitated. The age of the oldest embryos were sacrificed at ED14.

For further tissues implantations, all surgery was performed under Ketamine and Xylazine anesthesia, and all efforts were made to minimize suffering.

### Re-associations and *in vitro* culture

The dental epithelium and mesenchyme of ED14 mouse lower molars were dissociated by using 0.25% trypsin (BD Bioscience, Pont de Claix, France) and 1.2 U/mL dispase (Roche, Penzberg, Germany) in DMEM-F12 (Invitrogen, Villebon-sur-Yvette, France) (preheated to 37°C) at room temperature for 15 min. After separation of the dental epithelium from mesenchyme, each tissue was further dissociated into single cells. These were passed through a 70 µm nylon and pelleted by centrifugation at 9000 g for 2 min. The pellets containing mesenchymal and epithelial single cells were cut into fragments, re-associated and cultured on a semi-solid medium. This culture medium consisted of DMEM-F12 containing 20% FBS (PAA, Les Mureaux, France), 0.10 mg/mL of ascorbic acid (Merck, Lyon, France), 2 mM of L-glutamine (Invitrogen, Villebon sur Yvette, France), 50 U/ml of penicillin/streptomycin (Invitrogen, Villebon sur Yvette, France), and 0.36% of agar (Sigma-Aldrich, Lyon, France). Cultures were performed at 37°C in a humidified atmosphere of 5% CO_2_ for 8 days. The medium was changed every two days [17]. The trigeminal ganglia (TG) were dissected from ICR mice (PostNatal day (PN) 0 to 3) under a stereomicroscope (Leica MZ95, Nanterre, France) and co-cultured with dental cell re-associations overnight on semi-solid medium, just before implantation.

### *In vivo* implantation

The dental cell re-associations were cultured for 7 days and further co-cultured overnight with trigeminal ganglia (TG) before implantation between skin and muscles, behind the ears of ICR (152 samples) (Charles River Laboratories, l′Arbresle, France), CsA-treated ICR (229 samples) or Nude (NMRI-nu/nu, Janvier Labs, Saint Berthevin, France) (98 samples) adult mice [2]. Cell re-associations were also implanted without trigeminal ganglia and used as controls (42 samples in ICR, 55 samples in CsA-treated ICR and 28 samples in Nude adult mice). The mice were anaesthetized by intraperitoneal injection of 100 mg/g of ketamine (Virbac, Centravet, Nancy, France) and 10 mg/g of Xylazine (Rompun® 2%, Centravet, Nancy, France). The implantations were maintained *in vivo* for one or two weeks. Then, implanted mice were sacrificed by lethal injection of pentobarbital (Centravet, Nancy, France) and the implants were harvested for either histological analysis, or immunostaining, or transmission electron microscopy (TEM).

### Histology

For histology, samples were fixed for 24 h in Bouin-Hollande and embedded in paraffin. Serial sections (7 µm) were stained with Mallory's stain. After long-term implantations, the samples were demineralized in 15% EDTA for 48 h.

### Fixation protocols and immunofluorescence

After skin removal, heads from PN3, PN4, PN7 and PN10 ICR mice were fixed for 6 h in 4% paraformaldehyde at 4°C. Then, the heads were immersed overnight in PBS containing 5% sucrose at 4°C, and then for 6 h in PBS containing 20% sucrose at 4°C. Before immunostaining, mouse heads at PN7 and PN10 were demineralized in 15% EDTA for 2 weeks. Finally, the samples were embedded in Tissue-Tek® OCT (Agar Scientific, Saclay, France) and frozen at −20°C overnight and then at −80°C. Dissected ED14 molars, trigeminal ganglia, as well as cultured and implanted samples were washed in PBS, mounted in Tissue Tek® (OCT) and frozen. All frozen samples were stored at −80°C before serial sectioning (10 µm) on a cryostat (Leica, CM3000).

Serial sections were rinsed with PBS and fixed for 10 min with 4% paraformaldehyde at 4°C. After washing three times for 5 min in PBS at room temperature, tissue sections were incubated for 30 min at room temperature in a blocking solution of 1% bovine serum albumin (BSA) and then incubated for 2 h with the primary antibodies at room temperature. Blood vessels were stained using rat monoclonal anti-mouse CD31 (1/200, BD Pharmingen, Evry, France) [18], rat monoclonal anti-mouse CD34 (1/100, Ozyme, Saint Quentin Yvelines, France), polyclonal goat anti-mouse CD146 (1/100, Santa Cruz, USA) or polyclonal rabbit anti-mouse α-SMA antibody (1/100, Abcam®, Cambridge, MA, USA). Nerve fibers were immunostained for the type III intermediate filament protein peripherin (polyclonal rabbit anti-mouse peripherin, 1/800, Abcam®, Cambridge, MA, USA). Ondotoblasts were immunostained using a polyclonal rabbit anti-mouse nestin antibody (1/50, tebu-bio, Le Perray en Yvelines, France). Schwann cells were immunostained by polyclonal goat anti-mouse S100 β chain antibody (1/100, Santa Cruz, USA). Following incubation, tissue sections were washed three times for 5 min each in PBS and incubated in the secondary antibody solution for 1 h at room temperature. Secondary antibodies were: donkey polyclonal anti-rat conjugated to Alexa 488 (1/200, Invitrogen, Villebon sur Yvette, France), donkey polyclonal anti-rabbit conjugated to Alexa 488 (1/200, Invitrogen, Villebon sur Yvette, France), donkey polyclonal anti-goat conjugated to Alexa 488 (1/200, Invitrogen, Villebon sur Yvette, France) and donkey polyclonal anti-rabbit IgG conjugated to Alexa 594 antibodies (1/500, Invitrogen, Villebon sur Yvette, France). Negative controls were performed with corresponding sera instead of the primary antibody. After secondary incubation, sections were washed three times for 5 min each in PBS at room temperature. Slides were mounted in fluorescence mounting medium (Dako, Trappes, France) and observed with a microscope (Leica DM4000B) equipped for fluorescence.

### Transmission Electron Microscopy

The samples were fixed by immersion in 2.5% glutaraldehyde and 2.5% paraformaldehyde in cacodylate buffer (0.1 M, pH 7.4), demineralized in 15% EDTA for 2 weeks, and post-fixed in 1% osmium tetroxide in 0.1 M cacodylate buffer for 1 h at 4°C and dehydrated through graded alcohol (50, 70, 90, 100%) and propylene oxide for 30 min each under agitation. Samples were embedded in Epon 812. Semi-thin sections were cut at 2 µm with an ultra microtome (Leica Ultracut UCT) and stained with toluidine blue, and histologically analyzed by light microscopy. Ultrathin sections were cut at 70 nm and contrasted with uranyl acetate and lead citrate and examined at 70 kv with a Morgagni 268 D electron microscope. Images were captured digitally by Mega View III camera (Soft Imaging System).

## Results

### Attempts to innervate bioengineered teeth implanted in ICR mice

**Figure 1:**
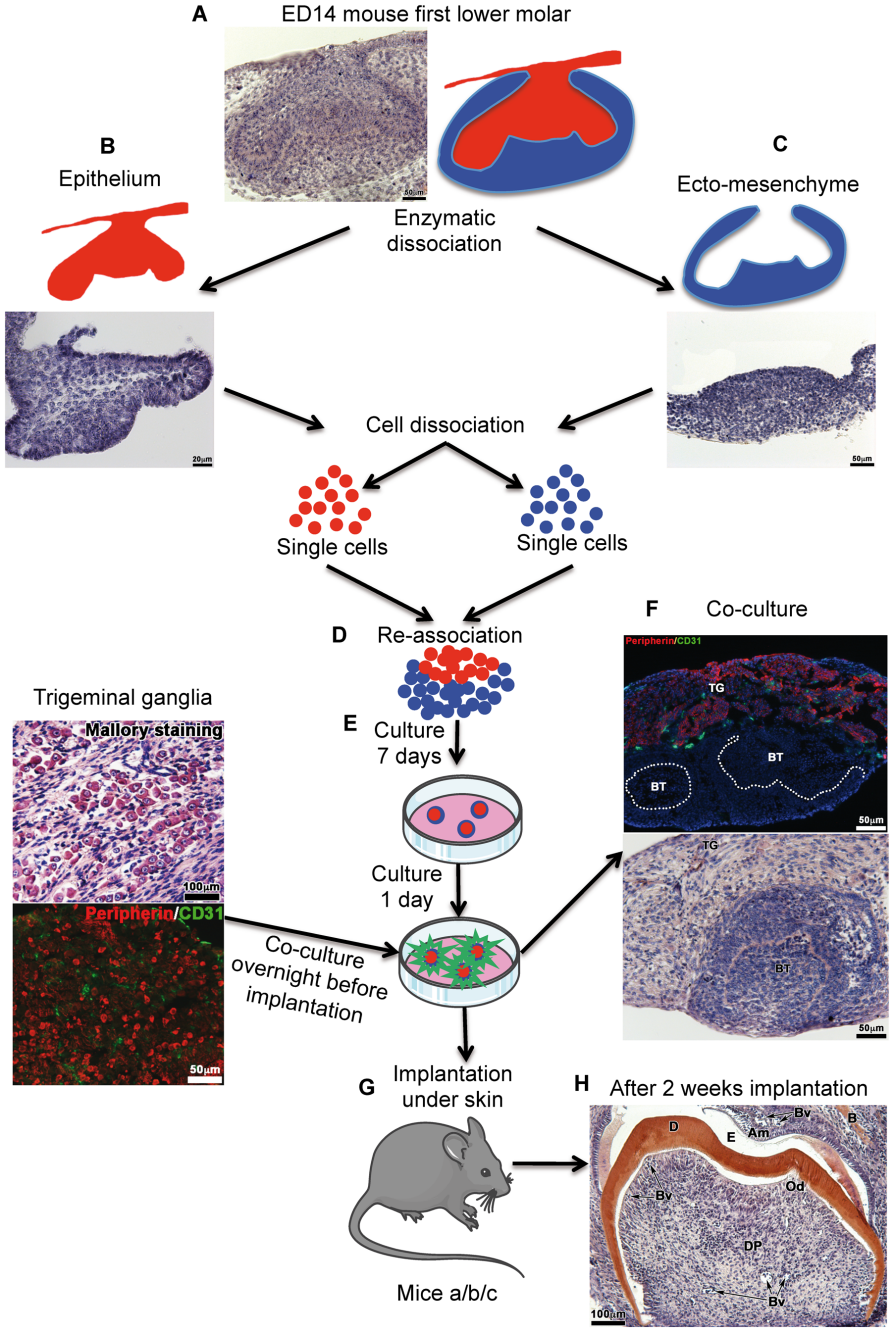
Protocol for tooth organ engineering. The mandibular first molars were dissected from ICR mouse embryos at embryonic day (ED) 14 (cap stage) **(A)**. Then, the dental epithelium **(B)** and ecto-mesenchyme **(C)** were separated by using a mixture of 0.25% trypsin and 1.2 U/mL dispase in DMEM-F12 (preheated to 37°C) at room temperature during 15 min. Each tissue was dissociated into single cells, which were then re-associated **(D)** and grown on semi-solid cultured medium **(E)**. After 7 days *in vitro*, each re-association was co-cultured overnight with trigeminal ganglia from ICR newborn mice **(F)**. The eighth day **(G)**, bioengineered tooth unit and trigeminal ganglia were co-implanted between skin and muscles behind the ears in adult ICR mice (mice a), CsA-treated ICR mice (mice b) and Nude mice (mice c) for 1 week or 2 weeks **(H)**. BT, bioengineered tooth; TG, trigeminal ganglia.

**Figure 2:**
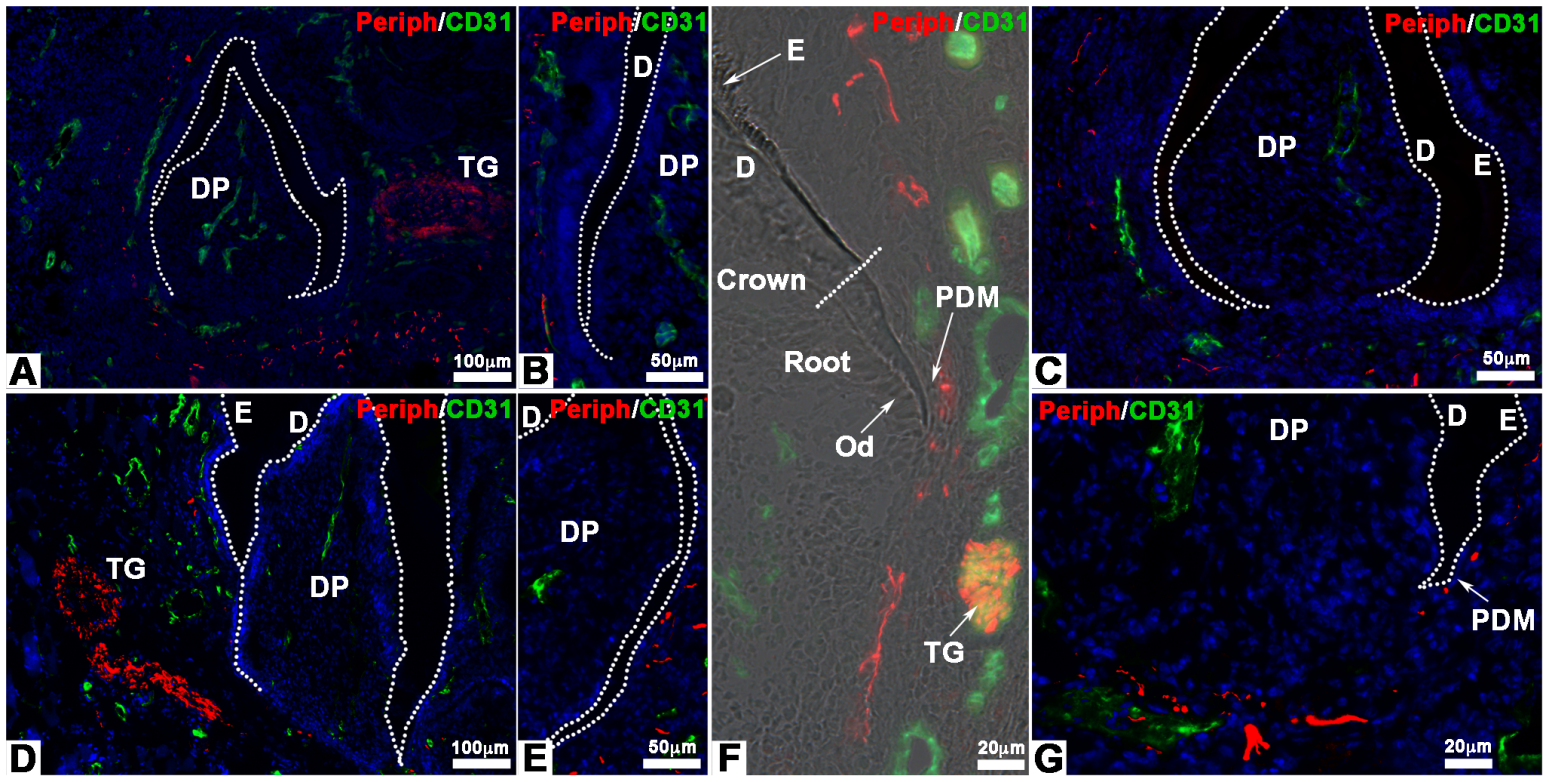
Innervation of bioengineered teeth implanted in ICR mice. Bioengineered teeth germs were co-implanted with trigeminal ganglia in adult ICR mice **(A–G)** for 1 **(A–C)** or 2 weeks **(D–G).** Nerve fibers and blood vessels in dental pulp and peridental tissues of bioengineered tooth were analysed immunohistochemically by using specific antibodies for peripherin (red) and CD31 (green). Blood vessels were present in peridental tissues and could enter in the dental pulp and reach odontoblasts already after 1 week of implantation **(A–C)**. Nerve fibers were detected in peridental tissues, in peridendal mesenchyme **(F)** and dental pulp but never in the dental pulp after 1 week **(A–C)** or even 2 weeks **(D–G)** of implantation. D, dentin; DP, dental pulp; E, enamel; Od, odontoblasts; PDM, peridental mesenchyme; TG, trigeminal ganglia.

Cultured cell re-associations have been implanted alone (42 re-associations) or co-implanted with trigeminal ganglia (152 re-associations) for one or two weeks under the skin of ICR adult mice (Fig. 1). In these conditions the crown formed and root development was initiated (Fig. 1H). Double immunostainings were performed using antibodies against peripherin to detect nerve fibers and against CD31 to visualize blood vessels (Fig. 2). Although re-associations were fully vascularized (Figs. 2A–G), nerve fibers originating from the host never entered the dental mesenchyme (Fig. 1I in [1]), neither after one week of implantation (0/18 samples) nor after two weeks (0/24 samples). Even when cell re-associations were implanted together with trigeminal ganglia, nerve fibers did not enter the dental pulp after one week (Figs. 2A–C; 0/29 samples). In most cases (120/123 samples) after two weeks of implantation, the results remained negative (Figs. 2D–G). Nerve fibers extended only in the tissues surrounding the forming tooth (Figs. 2A–G) and reached the limit between the peridental mesenchyme and the dental pulp (Fig. 2F, G). Only for 3 of the 123 re-associations co-implanted with trigeminal ganglia for two weeks, immunostaining for peripherin was positive in the dental mesenchyme (2,44%).

### Innervation of bioengineered teeth implanted in cyclosporin treated ICR mice

**Figure 3:**
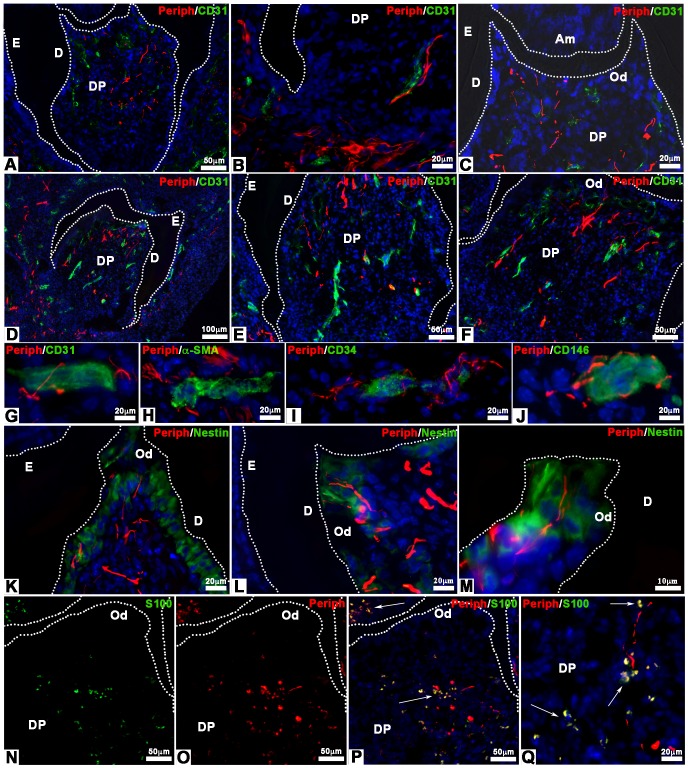
Innervation of bioengineered teeth implanted in cyclosporin A-treated ICR mice. Bioengineered teeth germs were co-implanted with trigeminal ganglia between skin and muscles behind the ears in adult CsA-treated ICR mice for 1 week **(A–C)** or 2 weeks **(D–Q)**. After transplantation, bioengineered teeth were analysed immunohistochemically by antibodies for nerve fibers (peripherin, red) and blood vessels (CD31, green), which showed that nerve fibers and blood vessels entered in the dental pulp after 1 and 2 weeks **(A–F)**. Double staining for peripherin (red) **(G–J)**, CD31 (green) **(G)**, α-SMA (green) **(H)**, CD34 (green) **(I)** and CD146 (green) **(J)** showed associations between nerve fibers and blood vessels in the dental pulp. After 2 weeks of implantation, odontoblasts were stained by an anti-nestin antibody (green) and nerve fibers by an anti-peripherin antibody (red) **(K–M)**. In this case, nerve fibers reached the odontoblast layer **(K–M)**. Immunofluorescence detection of S100 protein (green) **(N, P, Q)** and peripherin (red) **(O, P, Q)** on 2 weeks implanted bioengineered teeth showed the presence of Schwann cells and nerve fibers, respectively, in the dental pulp. The overlapping of the S100 protein and peripherin immunoreactivity appeared yellow in the merged images **(P, Q)**. Am, ameloblast; D, dentin; DP, dental pulp; E, enamel; Od, odontoblast.

ICR mice were treated with CsA, an immunosuppressant, to check possible effects on tooth tissue innervation. Cultured re-associations were co-implanted with trigeminal ganglia for one week (41 samples) or two weeks (188 samples) under the skin of CsA-treated ICR mice (Fig. 3). After one week, nerve fibers were detected in the peridental tissues as well as in the dental pulp (Fig. 3A; 36/41 samples, 88%). Nerve fibers and blood vessels were found in close contact in the apical part of the dental pulp (Fig. 3B). Already after one week, nerve fibers and blood vessels had reached the odontoblast layer (Fig. 3C). The same situation was maintained after two weeks of implantation (Fig. 3D; 172/188 samples, 91.5%).

After two weeks, CD31, CD34 and CD146 positive blood vessels were localized all over the dental pulp (Figs. 3G, I, J), whereas α-SMA positive blood vessels were localized only in the lower part of the dental pulp (Fig. 3H). Complexes between nerve fibers and blood vessels were formed with immature blood vessels, positive for CD31 (Fig. 3G) and CD34 (Fig. 3I) all over the dental mesenchyme. Nerves were also found in the vicinity of mature blood vessels, surrounded by pericytes, which were positive for α-SMA (Fig. 3H) and CD146 (Fig. 3J). Blood vessels and nerve fibers were observed in close vicinity in the apical and central part of the dental pulp (Fig. 3E).

After two weeks of implantation, nerve fibers and blood vessels were present in the odontoblast layer (Fig. 3F). Odontoblasts were characterized by immunostaining for nestin (Fig. 3K), a specific marker for differentiated odontoblasts [19], [20]. Nerve fibers were present between odontoblasts and in contact with dentin (Figs. 3L, M). Double staining was performed for peripherin and S100 protein (Figs. 3N–Q), a Schwann cells marker (Fig. 3N). The merged images showed the presence of Schwann cells in the peridental tissues (Fig. 3P) and in the dental pulp (Figs. 3P, Q). Innervation of cultured cell re-associations was possible in CsA-treated ICR mice when co-implanted with trigeminal ganglia of newborn ICR mice.

### Innervation of bioengineered teeth implanted in CsA-treated ICR mice by transmission electron microscopy

**Figure 4:**
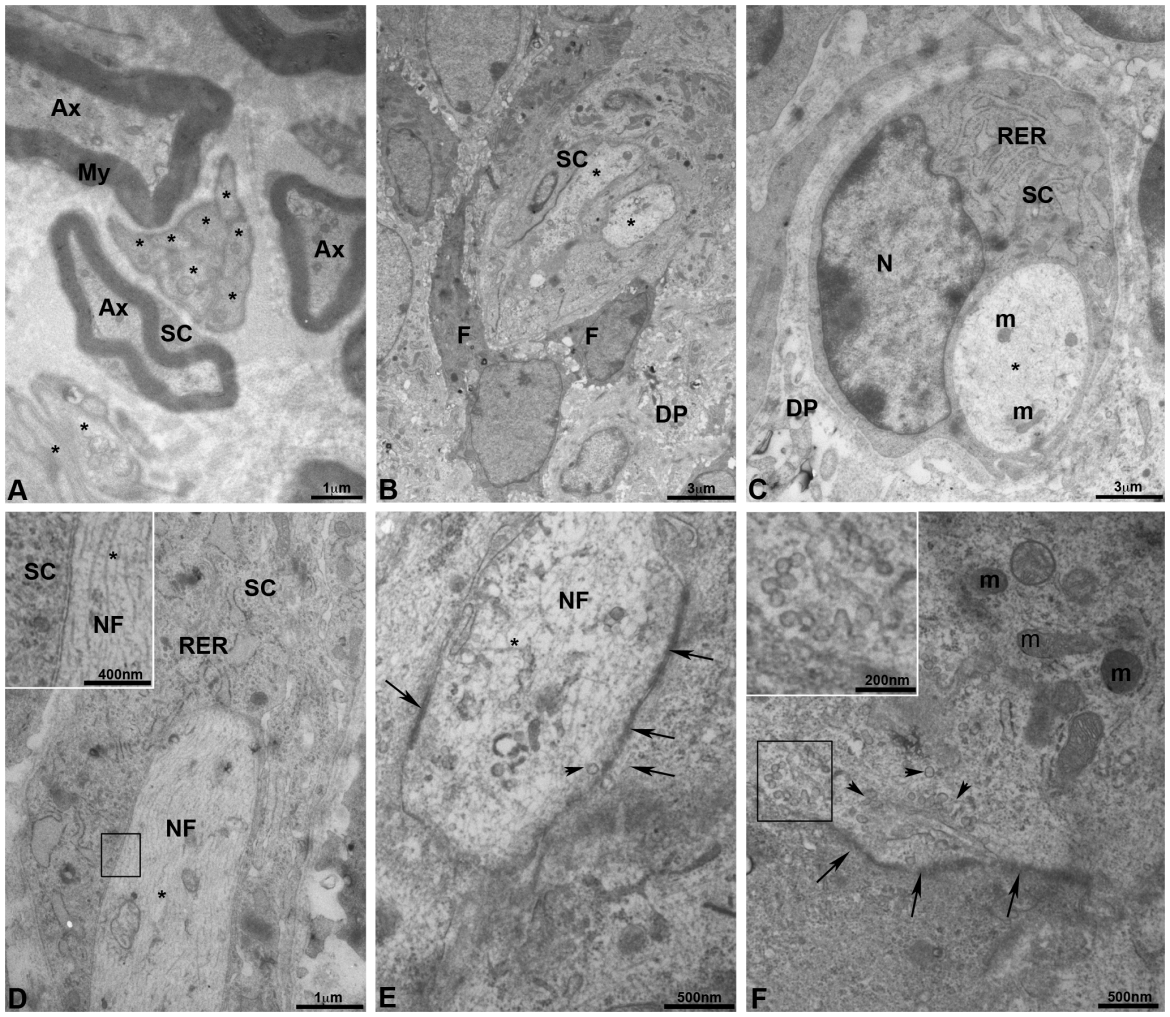
Innervation of bioengineered teeth implanted in cyclosporin A-treated ICR mice by transmission electron microscopy. Transmission electron microscopy (TEM) of trigeminal ganglia **(A)** showed the presence of myelinated and unmyelinated axons surrounded by Schwann cells **(A)**. TEM of dental pulp of epithelial and mesenchymal cell-cell re-associations co-implanted for 2 weeks with trigeminal ganglia in CsA-treated ICR mice showed the presence of unmyelinated axons **(B)**. These axons were surrounded by a Schwann cell and located near fibroblasts, which secreted collagen **(B)**. In **C**, one unmyelinated axon was surrounded by a Schwann cell with a developed rough endoplasmic reticulum. Neurofilaments **(D, E)**, numerous secretory vesicles **(arrowheads in F and insert)** and mitochondria **(F)** were present in the axons. A typical structure of a pre-synapse with numerous mitochondria and synaptic vesicles **(arrowheads)** was observed **(F)**. Thickening of the membrane suggested presence of synaptic contacts **(arrows in E and F)**. Ax, myelinated axon; DP, dental pulp; F, fibroblasts; m, mitochondria; N, nucleus; NF, neurofilaments; My, myelin; RER, rough endoplasmic reticulum; SC, Schwann cells; *, unmyelinated axon.

Myelinated and unmyelinated axons, separated by a collagen-rich extracellular matrix, were present in trigeminal ganglia. This result was also observed after two weeks of co-implantation with dental cell re-associations (Fig. 4A). Myelinating Schwann cells formed a compact mutilayered sheath around large diameter axons (Fig. 4A) and non-myelinating Schwann cells ensheated several axons of smaller diameter (Fig. 4A). In the pulp of teeth coming from implanted cell re-associations in CsA-treated ICR mice, only unmyelinated axons were detected (Figs. 4B–F). Schwann cells (Figs. 4B–D) showed a developed rough endoplasmic reticulum (Figs. 4C, D). In axons, neurofilaments (Figs. 4D and insert, 4E), mitochondriae (Figs. 4C, F) and synaptic vesicles (Figs. 4E, F) were observed by TEM. Thickenings of the plasma membrane suggested presence of synaptic contacts (Figs. 4E, F).

### Bioengineered teeth implanted for two weeks in CsA-treated ICR mice: morphological data

**Figure 5:**
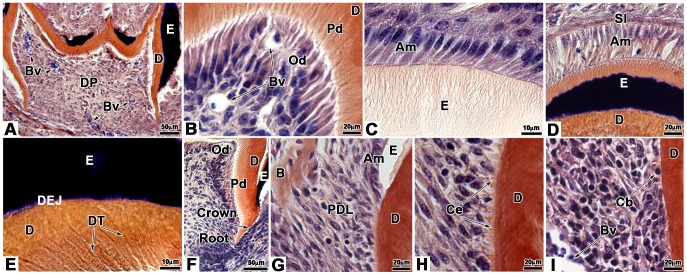
Histology of bioengineered teeth implanted for 2 weeks in cyclosporin A-treated ICR mice. Bioengineered teeth implanted for 2 weeks under the skin in CsA-treated ICR mice were analysed by histology. Crown development was identical to that observed for Nude mice **(A)**. Blood vessels could enter in the dental pulp **(A)**, migrated in the pulp **(A)** and reached odontoblasts **(B)**. Odontoblasts were elongated and polarized by the position of the nucleus, opposite to the secretory pole **(B)**. Thus the secretion of predentin/dentin was polarized **(B)**. As well as to Nude mice, ameloblasts of bioengineered teeth implanted in CsA-treated ICR mice, were elongated, polarized and secreted enamel **(C)**. In contact with ameloblasts stratum intermedium could be observed **(D)**. In the predentin/dentin, dentinal tubules were present and reached the dentin-enamel junction **(E)**. After 2 weeks of implantation, root was developed **(F)** and newly formed bone was present in dental mesenchyme **(G)** as well as in Nude mice. In contact with the external surface of dentin, cementoblasts were observed **(I)**. These cells were functional and deposited cementum **(H)**. Periodontal ligament was attached to the root by cementum and extended until reaching newly formed bone **(G, H)**. Am, ameloblast; B, bone; Bv, blood vessel; Cb, cementoblast; Ce, cementum; D, dentin; DEJ, dentin-enamel junction; DP, dental pulp; DT, dentinal tubule; E, enamel; Od, odontoblast; Pd, predentin; PDL, periodontal ligament; SI, stratum intermedium.

**Figure 6:**
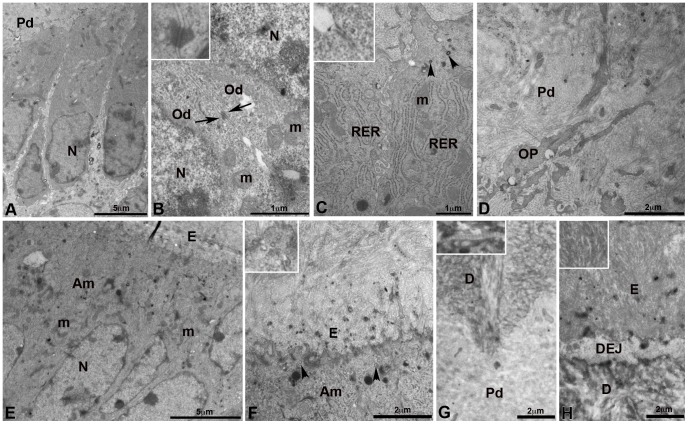
Transmission electronic microscopy of bioengineered teeth implanted for 2 weeks in cyclosporin A-treated ICR mice. Odontoblasts, dentinogenesis **(A–D, G)** and ameloblasts-enamel **(E, F, H)** in bioengineered teeth implanted for 2 weeks in CsA-treated ICR mice were analysed by transmission electron microscopy. Elongated odontoblasts showed a polarized position of the nucleus **(A)**. A desmosome was observed between two odontoblasts **(B: arrows and insert)**. In the cytoplasm of odontoblasts, the rough endoplasmic reticulum, mitochondria and secretory vesicles **(arrowheads)** were present in the supra-nuclear area **(C)**. The insert in **C** showed a zonula adherens between two odontoblasts. Odontoblast processes were surrounded by collagen fibers from predentin **(D)**. Ameloblasts were elongated and their nuclei were distant from the secretory pole **(E)**, which contained numerous vesicles **(F, arrowheads and insert)**. The junction between predentin and mineralized dentin was clearly visible **(G)**. The insert in **G** showed the typical periodic striation of collagen fibers. The dental-enamel junction showed typical organization of enamel **(E and insert)** and dentin **(H)**. Am, ameloblast; D, dentin; DEJ, dentin-enamel junction; E, enamel; Od, odontoblast; m, mitochondria; N, nucleus; OP, odontoblast processes; Pd, predentin; RER, rough endoplasmic reticulum.

The status of cell re-associations implanted for two weeks under the skin in CsA-treated mice was analyzed by histology (Fig. 5) and transmission electron microscopy (Fig. 6). As well as in untreated ICR mice [2], teeth developed, showing a well formed crown (Fig. 5A) and the initiation of root formation (Figs. 5A, F, G, H, I). Blood vessels were observed in the apical and central part of the dental pulp and reached the odontoblasts (Figs. 5A, B). Odontoblasts were elongated and showed a cytological polarization, as evidenced by the position of the nucleus, opposite to the secretory pole (Figs. 5B, 6A) and the localization of the rough endoplasmic reticulum (Fig. 6C). Different types of junctions between odontoblasts were observed including desmosomes (Fig. 6B and insert) and zonula adherens (Fig. 6C and insert). Odontoblasts also showed functional differentiation: development of the rough endoplasmic reticulum (Fig. 6C), presence of secretory granules (Arrowheads Fig. 6C), and polarized secretion of predentin/dentin (Figs. 5B, 6G). The mineralization of dentin occurred normally (Figs. 5A, 5B; 6G and insert, 6H). Numerous odontoblast cell processes were observed in predentin and dentin (Figs. 5B, 5E; 6D). Dentinal tubules in predentin/dentin reached dentin-enamel junction (Fig. 5E).

Ameloblasts were elongated, polarized (nuclei distant from the secretory pole) and functional with deposition of enamel (Figs. 5C, 5D, 6E). They were also in contact with flat cells of the stratum intermedium (Fig. 5D). Many coated vesicles were present at the secretory pole of ameloblasts (Fig. 6F and insert) suggesting a locally active re-internalization process. The enamel showed a typical crystal organization (Fig. 6H and insert).

Cementoblasts secreted the cementum on the external surface of root dentin, which allowed periodontal ligament cells to anchor in this matrix (Figs. 5H, G). These cells extended towards newly formed bone (Fig. 5F).

The development of teeth which formed after two weeks of implantation under the skin in CsA-treated ICR mice did not show any differences with those forming after implantation in ICR mice [2].

### Innervation of bioengineered teeth implanted in Nude mice

**Figure 7:**
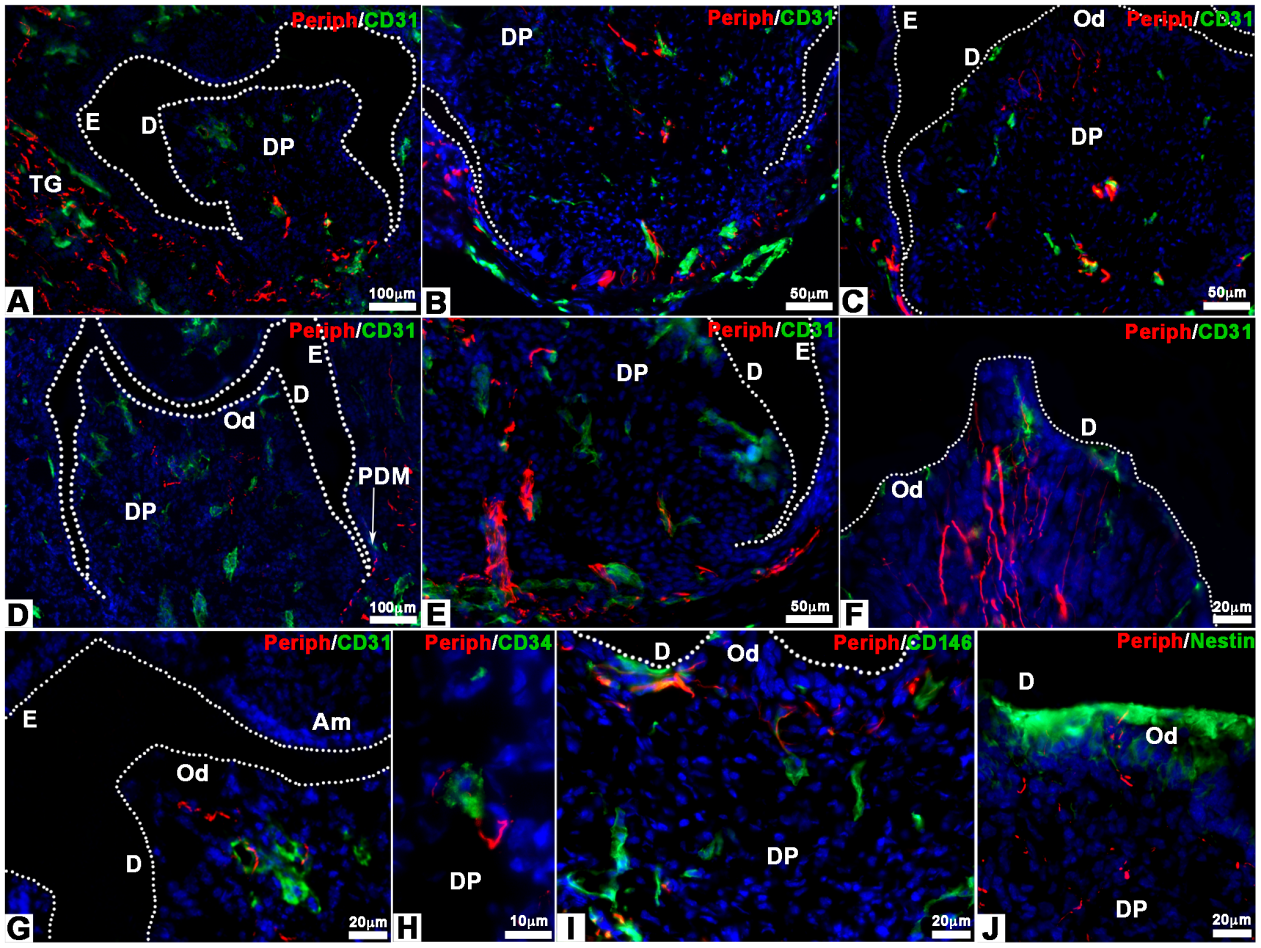
Innervation of bioengineered teeth implanted in Nude mice. Bioengineered teeth germs were co-implanted with trigeminal ganglia in adult Nude mice **(A–J)** for 1 **(A–C)** or 2 weeks **(D–J)**. Nerve fibers and blood vessels in dental pulp and peridental tissues of bioengineered teeth were analysed immunohistochemically by using specific antibodies for peripherin and CD31. Blood vessels were present in peridental tissues and could enter in the dental pulp and reach odontoblasts already after 1 week of implantation **(A–C)**. The staining for peripherin showed that nerve fibers entered in the dental pulp **(A, D)** and extend in the pulp **(B, E)** after 1 **(A–C)** and 2 **(D–J)** weeks. After 1 week of implantation, nerve fibers did not reach the odontoblasts **(C)**. This was achieved only after 2 weeks of implantation **(F, G, I, J)**. Double staining for peripherin **(G–I)**, CD31 **(G)**, CD34 **(H)** and CD146 **(I)** showed associations between nerve fibers and blood vessels in the dental pulp **(H)** and subodontoblastic layer **(G, I)**. After 2 weeks of implantation, nerve fibers, visualized by anti-peripherin antibody, had reached the odontoblast (positive for nestin) layer **(J)**. Am, ameloblasts; D, dentin; DP, dental pulp; E, enamel; Od, odontoblasts; PDM, peridental mesenchyme; TG, trigeminal ganglia.

Similar experiments have been performed in Nude mice: 98 re-associations with trigeminal ganglia and 28 re-associations without trigeminal ganglia have been implanted. In these conditions, nerve fibers were present in the mesenchyme around the tooth and also entered the dental pulp (Figs. 7A–F). This was already detected already after one week of implantation (Figs. 7A–C). The presence of nerve fibers in the dental mesenchyme was observed in almost 87% of the re-associations (n = 33/38 samples) after one week and this proportion were maintained after two weeks (90%, n = 54/60 samples). After one week of implantation, nerve fibers were observed in close contact with blood vessels in both the apical (Fig. 7B) and central (Fig. 7B) parts of the dental pulp. However, nerve fibers had reached the basal pole of odontoblasts (Fig. 7C). After two weeks, the nerve fibers in the dental mesenchyme (Figs. 7D, E) had extended to reach odontoblasts (Fig. 7F). After two weeks, CD31, CD34 and CD146 positive blood vessels were localized all over the dental pulp (Figs. 7G, H, I). Complexes between nerve fibers and immature blood vessels were detected after staining for CD31 (Fig. 7G) and CD34 (Fig. 7H) all over the dental mesenchyme. Nerves were also found in the vicinity of mature blood vessels positive for CD146 (Fig. 7I). After two weeks of implantation, nerve fibers and blood vessels were present in the odontoblast layer (Figs. 7F, G, I, J). Odontoblasts were characterized by immunostaining for nestin (Fig. 7J). Nerve fibers were present between odontoblasts and in contact with dentin (Figs. 7F, J). In the absence of trigeminal ganglia, the implantation of cell re-associations under the skin of Nude mice did not allow a spontaneous innervation of the implants (0/28 samples).

### Histology of bioengineered teeth implanted for two weeks in Nude mice

**Figure 8:**
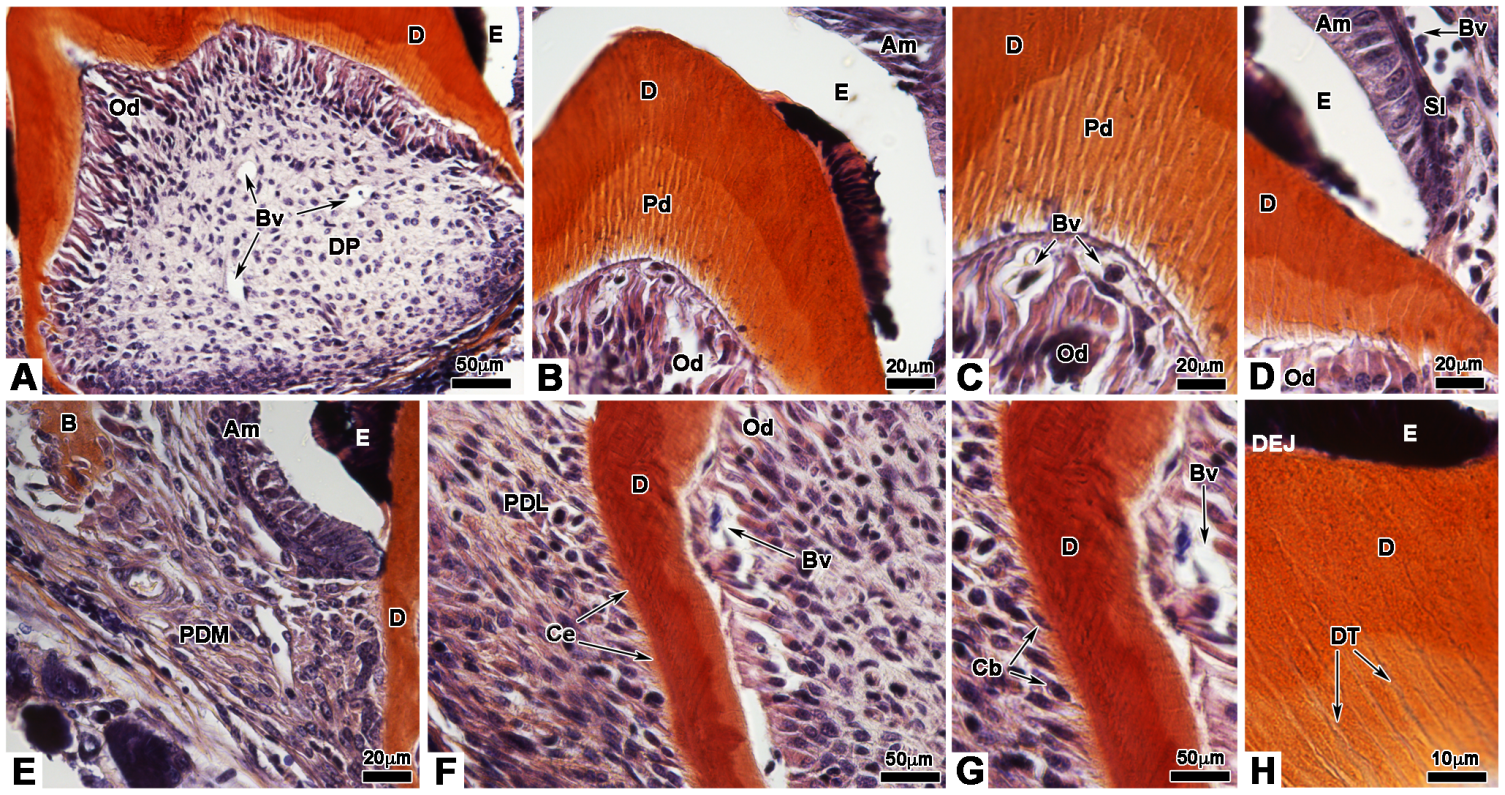
Histology of bioengineered teeth implanted for 2 weeks in Nude mice. After 2 weeks transplantation under the skin in Nude mice, bioengineered teeth germs were analysed by histology. The crown was well developed and blood vessels entered in the dental pulp **(A)**. Odontoblasts and ameloblasts became functional to secrete predentin/dentin and enamel respectively **(B)**. Odontoblasts were elongated and polarized **(C)**. Blood vessels migrated in the dental pulp and reached odontoblasts **(C)**. Elongated and polarized ameloblasts formed a monolayer in contact with the stratum intermedium where blood vessels could be found **(D)**. In the dentin, dentinal tubules extended toward the dentin-enamel junction **(H)**. Newly formed bone was present in peridental mesenchyme **(E)**. Below the crown-root junction, periodontal ligament were attached to the root by cementum and extended until reaching bone **(F)**, as shown in **(E)**. Cementoblasts were observed in contact with the external surface of dentin **(G)** and secreted the cementum **(F)**. Am, ameloblast; B, bone; Bv, blood vessel; Cb, cementoblast; Ce, cementum; D, dentin; DEJ, dentin-enamel junction; DP, dental pulp; DT, dentinal tubule; E, enamel; Od, odontoblast; Pd, predentin; PDL, periodontal ligament; PDM, peridental mesenchyme; SI, stratum intermedium.

Histology was performed to analyze the development of cultured cell re-associations implanted for two weeks in Nude mice and was compared it to implantations performed in ICR mice [2] and CsA-treated ICR mice (Fig. 5). After two weeks of implantation in Nude mice (Fig. 8), the crown was well formed (Fig. 8A). Odontoblasts were elongated and polarized: their nuclei showed an excentric position, opposite to the secretory pole (Figs. 8B, C). Odontoblasts were engaged in a functional differentiation by secreting predentin/dentin (Fig. 8B). Dentinal tubules were observed in predentin/dentin and extended toward the dentin-enamel junction (Fig. 8H). The presence of predentin/dentin allowed the induction of ameloblast cytological and functional differentiation (Fig. 8D). Ameloblasts were also elongated, polarized, and secreted enamel (Fig. 8D). In contact with ameloblasts, flatten cells formed the stratum intermedium (Fig. 8D). Blood vessels were observed, in contact with the stratum intermedium (Fig. 8D). Next to the crown-root junction, elongated mesenchymal cells were in contact with the acellular cementum on the root surface (Figs. 8F, G). These cells, corresponding to the principal periodontal ligament fibroblasts, extended towards newly formed bone (Fig. 8E). In the root, blood vessels were observed in close contact with functional odontoblasts (Figs. 8F, G). In contact with the external surface of root dentin, the cementum was secreted by the cementoblasts (Figs. 8F, G). The different steps of odontogenesis could take place and showed the same timing as observed after implantation in ICR mice and CsA-treated ICR mice (Fig. 5).

## Discussion

Previously published data have reported the innervation of engineered teeth after implantation in the jaw of adult mice [21]. However, and despite repeated attempts (n = 42), we could not observe any spontaneous innervation of the dental mesenchyme from cultured re-associations after implantation under the skin of adult ICR mice, thus confirming previous preliminary experiments [1]. The origin of the dental embryonic cells, the developmental stage of the teeth from which cells were prepared, and the methodology used to engineer teeth were very similar in the experiments performed in the two laboratories [17], [22]–[24]. Since the implantation under the skin, instead of jaw, might bring a limitation in the possibility of innervation, cell re-associations were implanted together with trigeminal ganglion, which mediates the sensory innervation of teeth and periodontal tissues in the lower jaw in physiological conditions [25]. In these conditions, neural growth was regularly observed extending from the trigeminal ganglion to all around the forming teeth. Although nerve growth did occur, the innervation of the dental mesenchyme was detected in less than 2.5% of samples (n = 152). There was a specific impairment of axons to enter the dental mesenchyme although, after two weeks of implantation, a thick layer of enamel has been deposited and axons should be able to reach odontoblasts [10]. After two weeks of implantation, the cell re-associations reach a stage corresponding to a first lower molar at PN4 when taking into account the crown development and stage of matrix deposition and mineralization [2], [26]. At PN4, axons can be detected in the dental pulp (Figs. S1D–G).

Previous works concerning face transplantation in human had shown that immunosuppressive therapy supported an unexpected reinnervation of the transplanted tissues [14]. Experimentally, the motor and sensory innervation of midface allotransplantation could be achieved in CsA-treated rats [27]. Cultured cell re-associations were thus implanted in ICR mice treated with CsA as an immunosuppressant. In these conditions, nerve fibers were detected in dental pulp already after one week. At this stage, nerves were even found to reach the odontoblast layer, which would correspond to PN7 *in vivo* (Figs. S1H–L). After two weeks, axons were present between odontoblasts and in contact with dentin, which would correspond to the physiological situation at PN10 (Figs. S1M–Q). Although only unmyelinated axons were detected, Schwann cells were present in both the peridental and dental mesenchymes of bioengineered teeth implanted in CsA-treated ICR mice. In physiological conditions, both myelinated and unmyelinated axons have been observed in the dental pulp of rodent molar [28].

Besides immunosuppression, CsA has been reported to interfere with growth cone activity, by stimulating their attraction [29]. Although these specific structures have not been searched for in implanted cell re-associations, growth cones have been described in the developing teeth in rodents [30], [31]. Other studies have shown that CsA stimulated axonal growth and induced GAP-43 expression [32]. Most dental axons express GAP-43 [33], [34]. Here may lay the main difference between our past experiments, where cell re-associations were implanted under the skin of adult untreated ICR mice [1], and those from the group of Tsuji, where cells re-associations have been transiently implanted in the kidney capsule using a CsA-treated mice [24], and then in the jaw of adult mice [21], [24]. However, these authors did not specify whether or not CsA-treatment was maintained during the implantation in the jaw [21]. Nevertheless, since CsA shows multiple effects (Fig. S2) [29], [32]–[39], including on T cells activity (Fig. S2A) and proliferation (Fig. S2A, B), as well as direct ones on nerve growth (Fig. S2C), attempts were made to check whether immunosuppression by itself could interfere with the innervation. To evaluate it, cell re-associations and trigeminal ganglia were co-implanted in immunodeficient Nude mice. In these conditions as well, the innervation of the dental mesenchyme was observed already after one week of implantation and axons reached the odontoblast layer after two weeks. The percentages of samples innervated after implantation in CsA-treated ICR and Nude mice were similar. This clearly showed that immunosuppression *per se* can interfere with and is sufficient to stimulate the innervation of the dental mesenchyme. Supportive data have recently been published, although in a very different experimental context. Indeed, the autologous transplantation of pulp stem cells with granulocyte-colony stimulating factor (G-CSF) in pulpectomized tooth was found to stimulate the regeneration of pulp tissue, including vascularization and innervation [40]. According to these authors, G-CSF together with conditioned medium of pulp stem cells, which stimulated cell migration and neurite outgrowth, also promoted immunosuppression *in vitro* [40]. Since in a clinical context, immunosuppressant therapy is a deep constraint and carries own risks, the possibility to use it temporarily only will have to be investigated [41].

Histological analysis and transmission electron microscopy were performed to ensure that the development of implanted cell re-associations in Nude mice or CsA-treated ICR mice did not show any alteration, when compared with re-associations implanted in control ICR mice [2]. Indeed, tooth crown morphogenesis, epithelial histogenesis and functional cell differentiation in both the crown and root areas progressed in a very similar way in all three models. After two weeks of implantation, odontoblasts were functional and accumulated predentin and dentin with characteristic organization as seen by transmission electron microscopy. Ameloblasts were polarized, secreted enamel, suggesting that the transport of calcium ions involved in enamel mineralization occurred normally. After two weeks of implantation, most ameloblasts already appeared as short cells with few rough endoplasmic reticulum cisternae, suggesting that they had already reached their maturation stage [42]. This reflected what happens during amelogenesis in mouse molars during development *ex vivo* and also when cell re-associations were implanted for two weeks in ICR mice without trigeminal ganglia [2]. The same was observed after co-implantation in ICR mice, Nude mice as well as in CsA-treated ICR mice. It is important to stress that when co-implantation was performed in CsA-treated mice, both the cytological and functional polarization of odontoblast and ameloblast did occur. All three constituents of the cytoskeleton (microtubules, microfilaments and intermediate filaments) are involved in these steps. CsA thus had no negative effect, although it is a specific inhibitor of the calcineurin-NFAT pathway [35]. Indeed, calcineurin is known to regulate the organization of the cytoskeleton [43]. On the other hand, calcineurin is strongly expressed in secretory odontoblasts and ameloblasts, where it was suggested to correlate with active mineralization phases [44]. Histology and transmission electron microscopy showed that there was no alteration in dentin and enamel secretion and mineralization when cell re-associations were implanted in CsA-treated ICR mice, instead of untreated ICR mice [2].

The results presented here show that the innervation of the dental mesenchyme, including the odontoblast layer, can be achieved in implanted re-associations of embryonic dental cells. However, this does not take place spontaneously but requires immunosuppressive conditions. It will then be important to check whether the different cellular partners of the network mediating sensory function as observed by Farahani et al. [9] in the human dental pulp can be found in implanted cell re-associations as they may exist in the mouse molar mesenchyme. Previous analysis of the cell heterogeneity in the dental mesenchyme of cultured and implanted re-associations showed that it can mimic the situation in the mouse first lower molars *in vivo* [26]. However, the different glial cell types and their network in implanted re-associations have not been investigated yet.

## Supporting Information

Figure S1 **Innervation of the dental mesenchyme of the first lower molar in ICR mice.** First lower molars from ICR mice at Postnatal (PN) days 3 **(A–C)**, 4 **(D–G)**, 7 **(H–L)** and 10 **(M–Q)** were analysed immunohistochemically for checking nerves development in the dental pulp. The goal was to have controls allowing comparison with the innervation as observed in experimental conditions, when cultured cell-reassociations were co-implanted with trigeminal ganglia. Nerve fibers (red staining) were visualized using an antibody directed against peripherin **(A–Q**) and relationships with blood vessels (green staining) using antibodies against CD31 **(A, B, H, I, M)**, CD34 **(B, E, K, P)** and CD146 **(C, F, G, L, Q)**. At PN3 **(A–C)**, the innervation of the dental pulp just started in its apical part **(A–C)**. Inserts, as magnification of boxes in **A** and **C** respectively, showed associations between nerve fibers and CD31 positive or CD146 positive blood vessels in the apical part of the dental pulp. However, such interactions could not found with CD34 positive blood vessels **(**insert in **B)**. At PN4 **(D–G),** nerve fibers had reached the central part of the dental pulp. Magnifications of boxed areas in inserts showed associations between nerve fibers and CD31 (**D**) or CD34 (**E**) positive blood vessels respectively. At this stage, nerve fibers were also associated with CD146 positive blood vessels **(G)**. At PN7 **(H–L)**, nerve fibers reached the basal pole of odontoblasts, characterized by their positive staining for nestin **(J)**. High magnifications from inserts in **H**, **K** and **L** showed associations between nerve fibers and CD31, CD34 and CD146 positive blood vessels in the central part of dental pulp. For CD31 positive blood vessels associations with nerve fibers were observed in the apical part of dental pulp **(I)**. At PN10 **(M–Q)**, nerve fibers were present in between odontoblasts **(O)** and peripherin was even detected in the dentinal tubules **(N)**. Associations between nerve fibers and CD31 positive blood vessels were observed in different parts of the dental pulp **(M)**, even very close from the odontoblast layer **(**boxed area and its magnification in insert in **M)**. Boxed areas and magnifications in inserts in **P** and **Q** showed associations between nerve fibers and CD34 (**P**) and CD146 **(Q)** positive blood vessels in the dental pulp. Av, alveolar nerve; Bv, blood vessel; D, dentin; DP, dental pulp; Od, odontoblasts.
(TIF)

Figure S2 **Mechanisms of action of cyclosporin A (CsA).** Three different mechanisms have been proposed in the literature **(A, B, C)**. **(A)** In the cytoplasm of T cells, CsA binds to cyclophylin (CpN) to form a complex. This complex binds and blocks the function of the enzyme calcineurin (CaN). Consequently, T cells do not produce some cytokines, which were necessary for full T cell activation. Furthermore, this pathway inhibits the proliferation of T cells. **(B)** Alternatively, CsA may increase transforming growth factor-beta1 (TGF-β1) transcription in interleukin-2 dependent T cells. This pathway also induces the inhibition of proliferation of T cells. In both cases **(A, B)**, the inhibition of T cells enhances axonal regeneration. **(C)** CsA increases the expression of growth associated protein-43 (GAP-43) expression in axonal growth cones and thus may have a direct effect on axonal extension. IL, interleukin; IFN-γ, interferon-gamma; GM-CSF, granulocyte macrophage-colony stimulating factor.
(TIF)
