# Supplementary figures and images for: Immunomodulation Stimulates the Innervation of Engineered Tooth Organ
Source: PLoS One. 2014 Jan 22;9(1):e86011. doi: 10.1371/journal.pone.0086011 (PMC3899083; doi:10.1371/journal.pone.0086011)

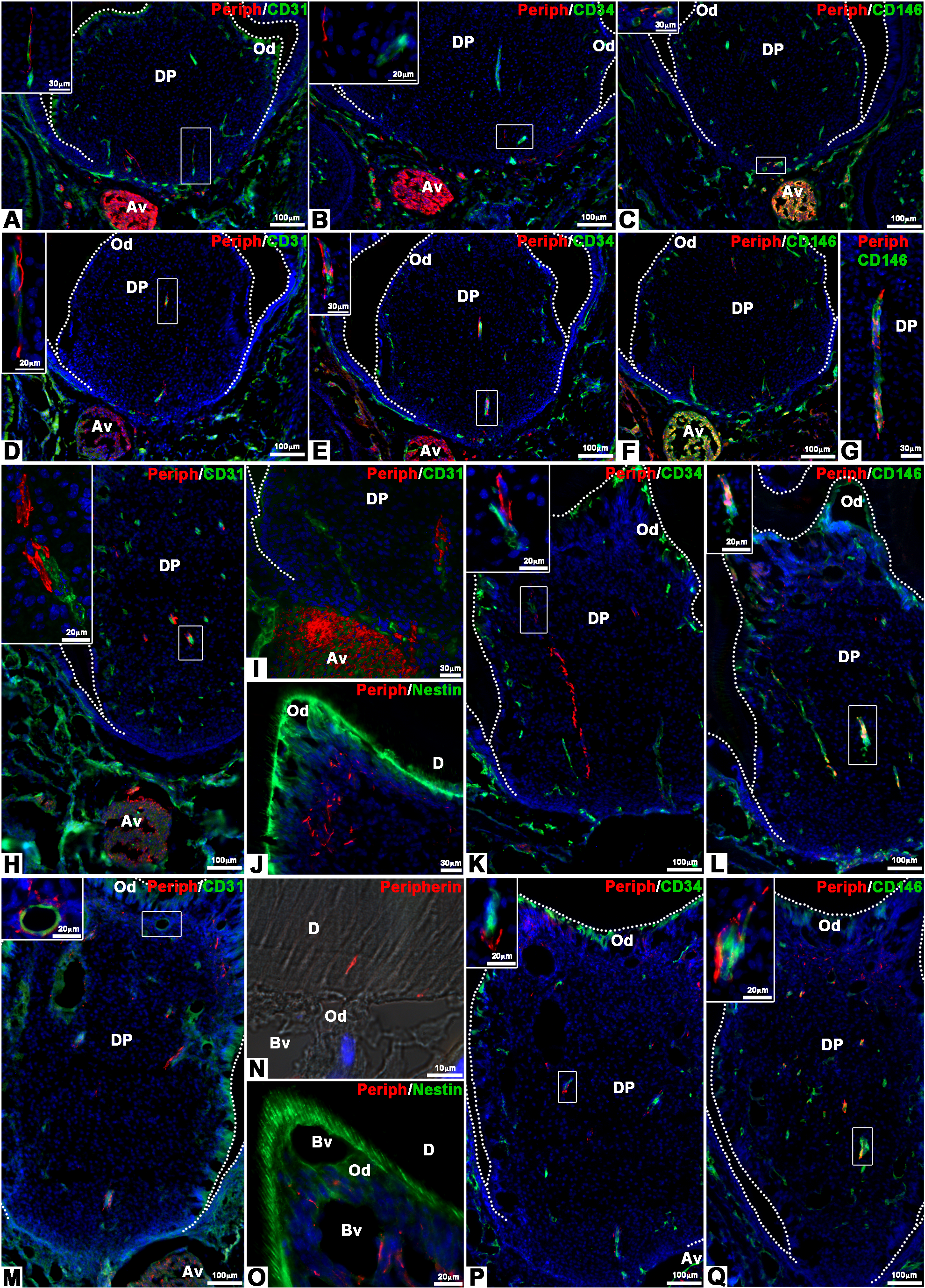

Supplement: Figure S1 — Innervation of the dental mesenchyme of the first lower molar in ICR mice. First lower molars from ICR mice at Postnatal (PN) days 3 (A–C), 4 (D–G), 7 (H–L) and 10 (M–Q) were analysed immunohistochemically for checking nerves development in the dental pulp. The goal was to have controls allowing comparison with the innervation as observed in experimental conditions, when cultured cell-reassociations were co-implanted with trigeminal ganglia. Nerve fibers (red staining) were visualized using an antibody directed against peripherin (A–Q) and relationships with blood vessels (green staining) using antibodies against CD31 (A, B, H, I, M), CD34 (B, E, K, P) and CD146 (C, F, G, L, Q). At PN3 (A–C), the innervation of the dental pulp just started in its apical part (A–C). Inserts, as magnification of boxes in A and C respectively, showed associations between nerve fibers and CD31 positive or CD146 positive blood vessels in the apical part of the dental pulp. However, such interactions could not found with CD34 positive blood vessels (insert in B). At PN4 (D–G), nerve fibers had reached the central part of the dental pulp. Magnifications of boxed areas in inserts showed associations between nerve fibers and CD31 (D) or CD34 (E) positive blood vessels respectively. At this stage, nerve fibers were also associated with CD146 positive blood vessels (G). At PN7 (H–L), nerve fibers reached the basal pole of odontoblasts, characterized by their positive staining for nestin (J). High magnifications from inserts in H, K and L showed associations between nerve fibers and CD31, CD34 and CD146 positive blood vessels in the central part of dental pulp. For CD31 positive blood vessels associations with nerve fibers were observed in the apical part of dental pulp (I). At PN10 (M–Q), nerve fibers were present in between odontoblasts (O) and peripherin was even detected in the dentinal tubules (N). Associations between nerve fibers and CD31 positive blood vessels were observed in different [file pone.0086011.s001.tif]

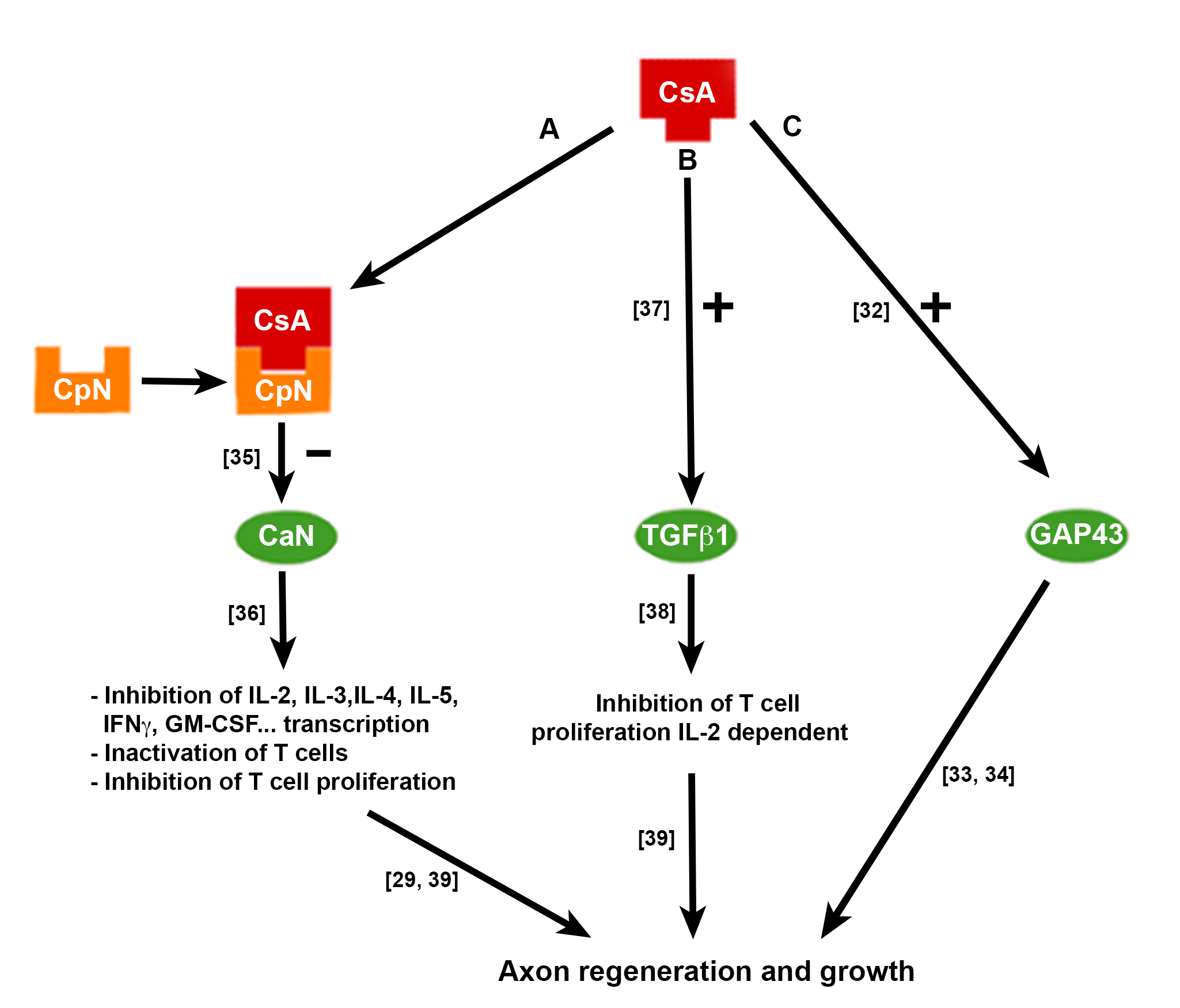

Supplement: Figure S2 — Mechanisms of action of cyclosporin A (CsA). Three different mechanisms have been proposed in the literature (A, B, C). (A) In the cytoplasm of T cells, CsA binds to cyclophylin (CpN) to form a complex. This complex binds and blocks the function of the enzyme calcineurin (CaN). Consequently, T cells do not produce some cytokines, which were necessary for full T cell activation. Furthermore, this pathway inhibits the proliferation of T cells. (B) Alternatively, CsA may increase transforming growth factor-beta1 (TGF-β1) transcription in interleukin-2 dependent T cells. This pathway also induces the inhibition of proliferation of T cells. In both cases (A, B), the inhibition of T cells enhances axonal regeneration. (C) CsA increases the expression of growth associated protein-43 (GAP-43) expression in axonal growth cones and thus may have a direct effect on axonal extension. IL, interleukin; IFN-γ, interferon-gamma; GM-CSF, granulocyte macrophage-colony stimulating factor. (TIF) [file pone.0086011.s002.tif]
